# Metallothionein 1: A New Spotlight on Inflammatory Diseases

**DOI:** 10.3389/fimmu.2021.739918

**Published:** 2021-11-05

**Authors:** Hanying Dai, Lu Wang, Lingyun Li, Zhong Huang, Liang Ye

**Affiliations:** ^1^ Department of Immunology, International Cancer Center, Shenzhen University Health Science Center, Shenzhen, China; ^2^ Respiratory Medicine Department, Shenzhen University General Hospital, Shenzhen, China

**Keywords:** metallothionein 1, inflammatory disease, signaling, metal, cytokine, immunoregulation

## Abstract

MT1 has been demonstrated to be an essential stress protein in maintaining physiological balance and regulating immune homeostasis. While the immunological involvement of MT1 in central nervous system disorders and cancer has been extensively investigated, mounting evidence suggests that MT1 has a broader role in inflammatory diseases and can shape innate and adaptive immunity. In this review, we will first summarize the biological features of MT1 and the regulators that influence MT1 expression, emphasizing metal, inflammation, and immunosuppressive factors. We will then focus on the immunoregulatory function of MT1 on diverse immune cells and the signaling pathways regulated by MT1. Finally, we will discuss recent advances in our knowledge of the biological role of MT1 in several inflammatory diseases to develop novel therapeutic strategies.

## Introduction

Metallothionein (MT) is a metal-binding protein with a low molecular weight and a high cysteine content found in all eukaryotes (1–3). The human *MT* gene is found on the q13 region of chromosome 16, whereas the mouse *MT* gene exists on chromosome 8. MT can be roughly divided into four subfamilies in humans, designated as MT1, MT2 (also known as MT2A), MT3, and MT4. MT1 genes have 18 isoforms, including 10 functional genes (*MT1A, MT1B, MT1E, MT1F, MT1G1, MT1G2, MT1H*, *MT1HL1*, *MT1M*, and *MT1X*) and 8 pseudogenes *(MT1CP, MT1DP, MT1JP, MT1L, MT1LP, MT1XP1, MT1P3*, and *MT1P1)*. The pseudogene isoforms of *MT2P1* and *MTL3P*, respectively, are found in MT2 and MT3 (3–5). MT1 and MT2 are ubiquitously expressed in almost all organs, especially in the kidney, liver, intestine, and pancreas. MT3 is present in a variety of organs, including the brain, the heart, the retina, the kidneys, the breasts, the prostate, the bladder, and the reproductive organs, whereas MT4 is found predominantly in the stratified squamous epithelium (6). Unlike the human MT, the mouse MT has only one functional gene for encoding each isoform (MT1, MT2, MT3, and MT4). MTs play an important role in regulating metal homeostasis and controlling physiological heavy metal toxicity, DNA damage, and oxidative stress (7–9). Since then, MTs have emerged as multiple effectors involved in immune homeostasis regulation (10–12). Accumulating experimental data from studies with MT-deficient mice or human samples has demonstrated the critical immunoregulatory role of MT isoforms in cancer, infectious diseases, central nervous system diseases, autoimmune diseases, and inflammatory bowel diseases. Notably, MT1 is widely expressed in almost every organ to maintain homeostasis. Therefore, it has been most extensively investigated in recent decades (4, 7, 10, 13–21).

Under physiological conditions, MT1 is involved in regulating the steady-state of metal, alleviating heavy metal poisoning, and protecting the body against oxidative stress, inflammation, and other cell damage caused by stress reaction (3). The immunoregulatory roles of MT1 in several inflammatory diseases derived from autoimmunity, infection, and inflammatory bowel diseases have been observed under pathological conditions. Elucidating the immunoregulatory functions and mechanisms of MT1 in inflammation diseases will provide a potent immunotherapy target for these diseases. In this review, we will highlight the immunomodulatory role of MT1 in inflammatory diseases that had initially been overlooked. We will first summarize the expression landscape of MT1 that different effectors trigger. We will then focus on important cellular targets of MT1, such as antigen-presenting cells (APCs), T cells, and basophils. We will also discuss the key molecular signaling pathways mediated by MT1. Finally, we will summarize the novel immunoregulatory effects of MT1 in various inflammatory diseases.

## The Inducers of MT1

As a metal transporter and antioxidative protein, MT1 exerts metal detoxification in organisms to maintain physiological element balance and prevent organ damage caused by an overload of metals. Many factors, such as heavy metals, antioxidants, alkylating agents, glucocorticoids, cytokines, and lipopolysaccharide, can trigger MT1 expression to produce a series of immunomodulatory effects.

### Metals/Antioxidants

Although metal ion regulation of MT expression has been reported in several reviews, MT1 induction by metal ions has not yet been systemically summarized. Zinc (Zn) is a transition metal in living organisms as a primary physiological inducer, identified as a crucial inducer of MT1. Previous studies found that ZnCl_2_-treated bone-marrow-derived dendritic cells (BMDCs) and human monocyte-derived DCs (moDCs) showed increased expression of MT1 on the cell surface. The membrane-bound MT1 present on ZnCl_2_-treated BMDCs can induce naive T cells to differentiate into regulatory forkhead box P3^+^ (FoxP3^+^) T cells by reducing levels of the costimulatory molecule CD86 and major histocompatibility class II (MHC-II) and by increasing production of the anti-inflammatory cytokine interleukin (IL)-10 and the tolerogenic DC marker immunoglobulin-like transcript 3 (ILT3). In contrast, MT1 deficiency in the zinc chloride (ZnCl_2_)-treated DCs was unable to induce a regulatory phenotype in T cells and failed to promote the proliferation of FoxP3^+^ T cells. Furthermore, zinc sulfate (ZnSO_4_) supplementation can induce tolerogenicity in DCs by increasing the expression of programmed death-ligand 2 (PDL2), cluster of differentiation 103 (CD103), and the enzyme indoleamine 2,3 dioxygenases, all of which are involved in regulatory T (Treg) cell differentiation (22). These findings imply that MT1 may program the phenotype and function of Zn-treated DCs to exert tolerogenicity (22, 23).

Ochratoxin A is a mycotoxin found in a variety of foods that causes oxidative stress and DNA damage. Supplement of ZnSO_4_ significantly enhances MT1 expression, which can protect against ochratoxin A induced-oxidative damage in human hepatocellular carcinoma (HepG2) cells (24). The association between Zn and MT1 was further supported by *in vivo* experiments, which discovered that dietary intake of Zn increased the expression of the *MT1* gene in the kidneys of rats (25). It has been demonstrated that zinc-induced *MT1* transcription is dependent on metal regulatory transcription factor 1 (MTF1) activation, which can be attributed to the abundant binding sites for MTF1 in the 5’ regulatory regions of *MT1* gene (26, 27). In turn, MT1 is required for Zn binding to regulate various cellular functions such as apoptosis, gene expression, differentiation, and proliferation.

In addition to Zn, copper (Cu), (Arsenic) As, chromium (Cr), and cadmium (Cd), and the phenolic antioxidant tert-butylhydroquinone (tBHQ) can induce MT1 expression. The mouse hepatoma cell line Hepa1c1c7 is highly responsive to toxic metal ions. When Hepa1c1c7 cells were exposed to the metals as mentioned above, MT1 was found to be highly expressed in the presence of Cu, As, Cr, Cd, or the phenolic antioxidant tBHQ (28). As previously discussed, MT1 expression confers a high capacity to bind these heavy metal ions *in vivo* and *in vitro*, thereby initiating the detoxification process (29).

### Inflammatory Factors

Acute-phase inflammatory responses can induce MT1 expression. It has been demonstrated that bacterial endotoxin-lipopolysaccharide (LPS) has an acute inductive effect on MT1 expression in a range of tissues, including the liver, heart, kidney, and brain. However, MT1 induction by LPS is rapid but not sustained and alters zinc metabolism, indicating that MT1 is a vital component of acute-phase inflammation (1, 30, 31). It should be noted that this enhancement effect occurs primarily in the LPS-sensitive CD1 strain of mice rather than the LPS-resistant C3H/HeJ strain of mice (32). Furthermore, acute inflammatory cytokines and double-stranded RNA (poly I: C) also effectively induce hepatic MT1 expression. Recombinant cytokines IL-1α, IL-1β, and interferon gamma (IFN-γ) can effectively induce MT1 expression in the ovary, uterus, and liver from LPS-sensitive CD-1 mice, whereas IL-6 and tumor necrosis factor alpha (TNF-α) were only effective in the liver (32). TNF-α is also a potent inducer of MT1 expression in the lung and heart, but IL-6 is unaffected by MT1 induction in either organs (33).

Although the molecular mechanism of induction of MT1 by acute inflammation is not entirely understood, intracellular second messengers may potentially explain the generation of MT1. DCs stimulated with LPS had lower intracellular free zinc, which increased major histocompatibility complex II (MHC-II) expression, whereas overexpression of the zinc transporter Zip6 could reverse this (22, 34). Supplementing Zn can impair surface MHC-II expression on DCs and suppress proinflammatory cytokine expression of interleukin (IL)-1β, IL-6, IL-12, and TNF-α (22). The evidence supports a hypothesis that MT1 expression induced by acute inflammatory factors may depend on the zinc transporter 6 (Zip6), altering intracellular zinc homeostasis.

In addition to intracellular zinc signaling, multiple promoter elements, such as signal transducer and activator of transcription (STAT) proteins, major late transcription factor/antioxidant response element (MLTF/ARE), glucocorticoid responsive element (GRE1), MTF1, and specificity protein 1 (SP1), may be involved in inflammatory factor-induced MT1 gene expression (35, 36). LPS administration can promote hepatic IL-6-dependent MT1 expression by STAT1 binding to the MT1 promoter (35). Similarly, STAT1 and STAT3 can bind to the MT1 promoter for initiating IL-6-induced MT1 and MT2 expression in response to influenza virus infection (36). Activation of STAT3 and STAT5 signaling pathways determines the levels of MT1 and MT2 expression in *Histoplasma capsulatum-*infected macrophages, and this process is also implicated in the Zn import involved in Zip2 regulation (37). Although Zip genes contain STAT binding sites (3), it is unclear whether STAT3 and STAT5 signaling directly impact Zip2 expression in these cells. Therefore, these findings suggest that inflammatory factors could activate multiple signaling pathways to drive MT1 expression, but the intracellular zinc environment may exist independently or coordinate with other intracellular signals.

### Immunosuppressive Factors

Dexamethasone, a widely prescribed immunosuppressive corticosteroid, has been identified as a critical inducer of MT1. Dexamethasone treatment can increase MT1 mRNA levels in various cell types, including DCs, HeLa cells, and Baby hamster kidney fibroblasts (BHK cells), and this effect can be boosted further by Zn supplementation (23, 38). Earlier studies indicated that the mouse MT1 and MT2 genes and human MT2 genes contain enhancer glucocorticoid response elements (GREs). Glucocorticoids, such as dexamethasone, can induce MT1 and MT2 expression *via* GREs (38–41). In humans, glucocorticoids significantly elevate the expression of MT2A over MT1 isoforms (38, 39).

The expression of MT1 induced by dexamethasone confers a tolerogenic DC phenotype. A previous study indicated that dexamethasone-treated DCs exhibited lower CD86, MHC-II, and glucocorticoid-induced leucine zipper (GILZ) expression on the cell surface and higher IL-10 levels (23). In contrast to Zncl_2_-treated DCs, total MT1 protein levels can be detected in dexamethasone-treated DCs but not on the cell membrane. Although supplementation of MT1 in activated T cells was able to enhance the percentage of FoxP3^+^ T cells, inhibiting MT1 only reduces the proportion of FoxP3^+^ T cells induced by Zncl_2_-treated DCs but not dexamethasone-treated DCs (23). These findings imply that dexamethasone-induced MT expression may preferentially serve as a marker of tolerogenic DCs but is not essential for tolerogenic DC function. The question of whether MT1-drive tolerogenic DC features are determined by zinc redistribution or direct interaction with other proteins on the cell membrane remains unanswered.

## Responsive Cells

### Antigen-Presenting Cells

**Figure 1:**
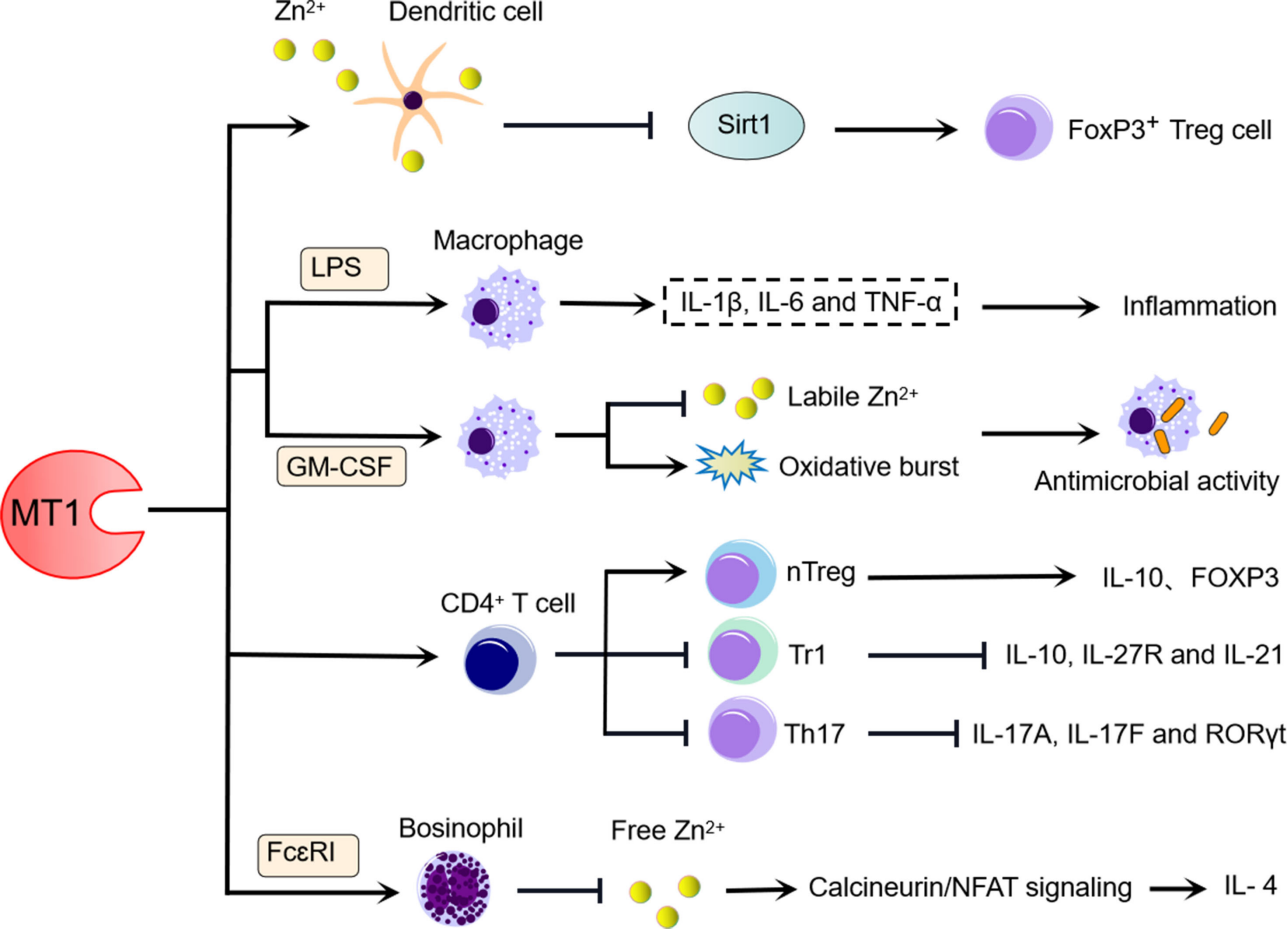
MT1 influences the differentiation and function of immune cells. (1) MT1 expressed on the cell membrane of IL-10-expressing DCs may dampen Sirt1-induced FoxP3 degradation and contribute to FoxP3^+^ T cell proliferation, and MT1 upregulation during this biological process is strongly dependent on ZnCl_2_ signaling. (2) High levels of MT1 in LPS-induced macrophages can facilitate inflammation, whereas the increased level of MT1 in GM-CSF-induced macrophage benefits the antimicrobial activity by inhibiting labile Zn^2+^ and increasing oxidative burst. (3) MT1 positively regulates the differentiation of CD4^+^ T cells towards Tregs and negatively modulates the differentiation of CD4^+^ T cells towards Tr1 and Th17. (4) MT1 promotes the production of IL-4 derived from FcεRI-induced basophils by limiting free Zn^2+^, which activates CaN/NFAT signaling.

Antigen-presenting cells (APCs), such as dendritic cells (DCs) and macrophages, serve as links between activation of innate and adaptive immunity and play a crucial role in governing T-cell immunity (42). APC functional dysregulation has the potential to initiate diverse immune diseases (43). Previous research has found that MT1 has some immunosuppressive properties towards DCs (**Figure 1**). IL-10-expressing DCs showed high levels of MT1, which is essential for inducing the T regulatory cell phenotype and promoting the proliferation of Foxp3^+^ T cells. Increased MT1 expression in IL-10-expressing DCs is dependent on dexamethasone or ZnCl_2_ stimulation, whereas upregulated MT1 expression on the DC membrane is strongly dependent on ZnCl_2_ but not dexamethasone stimulation. Furthermore, ZnCl_2_-treated DCs express MT1 on the cell surface, which preferentially induces the phenotype of Treg cells (23). Although the potential mechanism underlying this discovery is not yet fully clarified, it has been suggested that histone deacetylase Sirt1 is involved in the degradation of FoxP3 in Tregs. However, the presence of Zn can turn around the Sirt1-mediated degradation of FoxP3 in Treg (44). Thus, at least one possible mechanism is that MT1 on the surface of DCs donates Zn to Tregs, which in turn inhibits Sirt1-induced degradation of FoxP3 and maintains the phenotype of Tregs. Since FoxP3^+^ Tregs exhibit tolerance functions that play a crucial role in controlling inflammatory responses (45), further studies are required to determine the precise mechanism by which MT1-expressing DCs modulate FoxP3^+^ Tregs and to assess the role of MT1-expressing DCs in inflammatory diseases or autoimmune diseases.

In addition to their role as an APC, macrophages perform a variety of other functions in the fight against microbial invasions (37, 46). LPS stimulates macrophages to produce large amounts of proinflammatory cytokines such as TNF-α, IL-β, and IL-6, responsible for inflammatory damage and infection defense. MT1- and MT2-deficient macrophages dampen the expression of LPS-induced inflammatory cytokines, including TNF-α, IL-β, and IL-6. The enhanced effects of MTs on LPS-induced inflammatory responses in macrophages are directly due to the regulation of nuclear factor kappa B (NF-κB) activity. A recent report indicated that MT1 and MT2 were substantially elevated in granulocyte-macrophage colony-stimulating factor (GM-CSF)-activated macrophages in a STAT3- and STAT5-dependent manner during *H. capsulatum* infection. GM-CSF also alters Zn redistribution in *H. capsulatum*-infected macrophages by regulating transporters. Interestingly, macrophages lacking MT1 and MT2 recovered *H. capsulatum* survival, accompanied by labile Zn levels and dampened oxidative bursts (37). Thus, in the absence of MT1 and MT2, a lack of Zn sequestration in macrophages would reduce the ability of GM-CSF to inhibit *H. capsulatum* growth. Overall, these findings appear to support the importance of MT1 and MT2 acting on macrophages in defense against infection and inflammation *via* involving multiple signaling pathways (**Figure 1**).

### T Cells

As one of the suppressive T-cell subsets, type I regulatory T (Tr1) cells have been identified to regulate inflammation, graft-versus-host disease, and autoimmunity by producing IL-10 in response to IL-27 (47). IL-27 can induce MT1 expression in mouse and human Tr1 cells. Endogenous MT1 upregulation by IL-27 can suppress Tr1 cell differentiation by interfering with the IL-27-activated signaling pathway, which includes IL-27 receptor blockade and inhibition of IL-10 and IL-21. This inhibitory effect of MT1 on Tr1 cells can be supported further by using MT knockout mice deficient in both MT1 and MT2. IL-27 treatment of Tr1 cells increased the expression of IL-10 and upregulated the expression of IL-27R and IL-21 in MT-deficient mice. However, this abolished the phenotype of Tr1 cells, which can be compensated for by overexpression of either MT1 or MT2 (48). Thus, MTs negatively regulate Tr1 cell differentiation. In contrast to Tr1 cells, MT1 is a positive regulator of natural regulatory T (nTreg) cell differentiation (49). *In vitro*, MT1 can promote naive T cells toward nTreg cell differentiation. MT1-treated mice had a higher proportion of FoxP3^+^ Treg cells and higher levels of FoxP3 and IL-10 expression in Treg cells, confirming the positive regulatory effects of MT1 on nTreg cells (50) (**Figure 1**).

Notably, MT1 is highly expressed in T helper 17 (Th17) cells, but it has a negative regulatory role in Th17 cells (50). MT1 can suppress Th17 cell differentiation and inhibit the expression of Th17-related genes such as IL-17A and IL-17F *in vitro* (**Figure 1**). However, MT1 did not affect Th17 cell proliferation and apoptosis under Th17-skewing conditions (46). These findings demonstrate that MT1 can regulate T-cell differentiation programs by diverse potential mechanisms (**Figure 1**).

### Basophils

Although basophils represent <1% of the peripheral blood leukocytes, they exert an important immune regulatory role in allergic inflammation by releasing Th2 cytokines [IL-4 and thymic stromal lymphopoietin (TSLP)] and expressing cell-surface high-affinity IgE receptor (FcεRI) (51, 52). Recent studies have demonstrated the substantial immunoregulatory function of MT1 in basophils (**Figure 1**). As demonstrated by IgE-stimulated basophils, activation of either bone marrow-derived basophils (BMBAs) or primary basophils in response to FcεRI stimulation can effectively induce MT1 expression (53). Even though MT1 can promote the synthesis of IL-4 in basophils upon FcεRI stimulation, it did not affect the development of basophils. In contrast, basophils with MT1 deficiency showed low levels of IL-4 expression after FcεRI stimulation. The enhancement role of MT1 in basophils necessitates a novel molecular mechanism that involves maintaining intracellular Zn levels to regulate calcineurin activity and nuclear factor of activated T-cell (NFAT) signaling (53). These findings indicate that MT1 could be a valuable therapeutic target for basophil-mediated immune responses and allergic inflammation.

## The Main Regulatory Mechanisms of MT1

### MT1 Impairs the STAT Signaling Pathway

**Figure 2:**
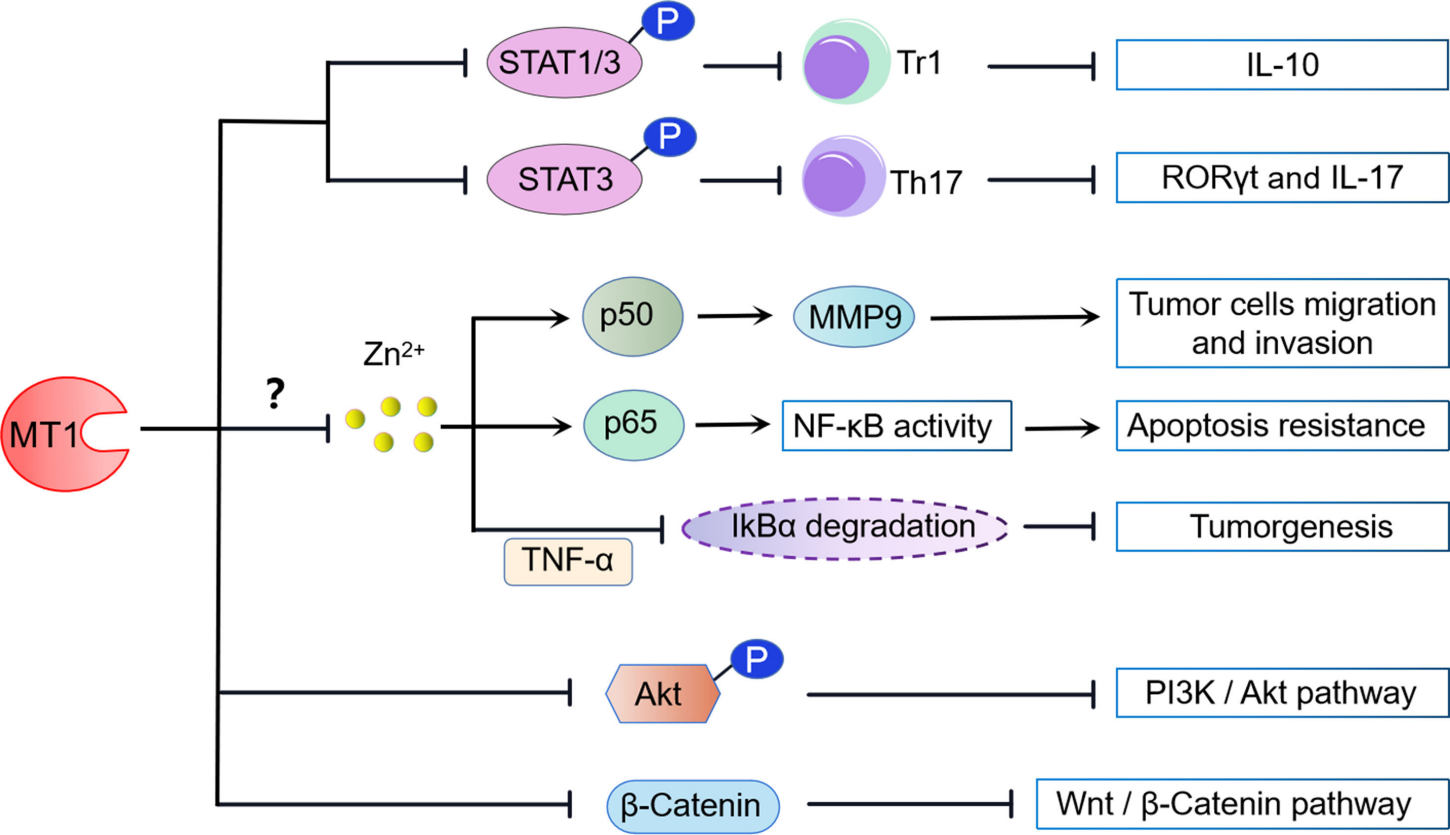
MT1 modulated signaling pathways. (1) MT1 impairs the phosphorylation of STAT1/3, preventing the differentiation of IL-10-producing Tr1 cells. MT1 also negatively regulates Th17 cells by inhibiting the phosphorylation of STAT3. (2) MT1 can positively modulate the NF-kB signaling pathway by the upregulation of NF-kB p50/P65 activity, contributing to tumor cell migration, invasion, and apoptosis resistance. In contrast, MT1 can also dampen tumorigenesis by negatively regulating TNF-α-induced degradation of IkBα. These biological activities may be aided by MT1-mediated free Zn^2+^ inhibition. (3) MT1 suppresses the PI3K/Akt signaling pathway by restraining Akt phosphorylation. (4) MT1 negatively modulates the Wnt/β-catenin pathway by limiting the nuclear translocation of β-catenin.

The functions of transcription factors STAT1 and STAT3 are tightly regulated, and both activation and inactivation of function lead to the occurrence of immune diseases (54, 55). MTs have been shown to play a role in regulating STAT1 and STAT3 signaling (**Figure 2**). Either MT1 or MT2 signaling inhibition in Tr1 cells resulted in STAT1 and STAT3 hyperphosphorylation and IL-10 production, indicating that MT1 and MT2 are negative STAT1 and STAT3 signaling regulators. In contrast, overexpression of either STAT1 or STAT3 could reverse the suppressive role of MT1 and MT2 in Tr1 cells. Moreover, MT1 and MT2 can prevent Tr1 differentiation and further potentially compete with positive regulators of IL-10 to achieve its inhibitory effect. Thus, activating the MT1/MT2-STAT1/3 axis may form a kinetic balance to control IL-10 production in Tr1 cells (48).

Meanwhile, MT1 also contributes to Th17 cells *via* regulating STAT3 signaling. Our previous study found that MT1 treatment abolished the STAT3 phosphorylation in CD4^+^ T cells under Th17-skewing conditions. Consistently, the transcription factors and Th17-related genes, retinoic acid-related orphan receptor-γt (RORγt), and IL-17 were also markedly reduced in the Th17 cells under MT1 stimulation. Furthermore, administration of an adenovirus expressing MT1 in mice decreased STAT3 phosphorylation and RORγt expression in synovial tissues, which attenuated rheumatoid arthritis symptoms (50). Collectively, these results indicate that MT1 modulates the differentiation and function of Th17 cells, which is attributed to the suppression of the STAT3 signaling pathway (**Figure 2**).

### MT1 Dually Regulates the NF-kB Signaling Pathway

Nuclear factor-kappa B (NF-κB) is a transcription factor typically composed of two protein subunits, p50 and p65, and is usually found in the cytoplasm bound to an inhibitory IκB subunit. NF-κB regulates a massive number of genes involved in immune and inflammatory responses. MT1 may possess dual functions to participate in NF-κB activation (**Figure 2**). Loss of MT in fibroblastic cell lines diminished nuclear NF-kB p65 subunit but did not decrease NF-kB transcriptional activity, consistent with increased cell sensitivity to apoptosis. Reconstitution of MT1 expression in MT knockout cells restored NF-kB p65 subunit levels, increased NF-kB activity, and promoted resistance to apoptosis, suggesting that MT1 is a crucial positive regulator of NF-kB activity (56). Similar studies confirm that overexpression of MT in human breast carcinoma-derived cell lines promotes NF-kB binding to DNA and induces NF-kB-mediated gene expression (57). MT1 can also enhance the migration and invasion of human glioma cells by inducing matrix metallopeptidase 9 (MMP-9) activation through the upregulation of NF-kB p50 activity (58). However, other groups have reported contradictory effects of MT1 on NF-kB activity. Transfection of the mouse MT1 gene into MT-KO cells can suppress tumor necrosis factor-α (TNF-α)-induced NF-kB-dependent gene expression by blocking IkBα degradation (59). Furthermore, stable expression of the new functional member of MT1, MT1m, blocked TNF-α-induced degradation of IkBα and transactivation of NF-κB in human hepatocellular carcinoma. Thus, downregulation of MT1 might contribute to tumorigenesis by increasing cellular NF-κB activity (60).

Since MTs expression has been linked to zinc release to maintain physiological activity in various cell culture systems, MT1 may play a pivotal role in NF-κB signaling *via* zinc sequestration. Zinc and zinc ionophores displayed apparent inhibitory effects on NF-κB DNA binding activity in a dose-dependent manner in HeLa cells (61). This effect was also observed in monocytes, macrophages, endothelial cells, and lung epithelia (62, 63). Consistent with these findings, loss of zinc importer Slc39A8 (Zip8) resulted in increased IKKβ two substrates phosphorylation of P65 and IkBα (62). In contrast, loss of zinc exporter Slc30a5 (Znt5) in mast cells led to elevated labile zinc and inhibited NF-kB signaling (64). These results suggested that intracellular zinc is a potent negative regulator of NF-kB activity through inhibiting IKK kinase activity. Intriguingly, overexpression of MT-IIA or metal/hormone-induced MT expression counteracts the inhibitory effects of zinc on NF-kB binding activity (61), indicating that MT is required for zinc-mediated NF-kB signaling pathway modulation. Although the independent role of MT1 in controlling the axis of zinc/NF-kB is not completely clear, blockade of Zip8-mediated zinc transport remarkably restrains MT1 expression while enhancing NF-kB signaling (61). Therefore, these findings implicate MT1-Zn as one of the potential intracellular modulators of NF-kB activation.

### MT1 Suppresses the Wnt/β-Catenin and PI3K/Akt Signaling

In mice, there is a rapid upregulation of MT1/2 in response to intermittent hypoxia (IH)-induced endoplasmic reticulum (ER) stress. Deficiency of the MT1/2 gene exacerbates IH-induced ER stress and associated cell death, but it is entirely prevented by overexpression of MT1/2. The protective effects of MT1/2 against IH-induced ER stress and apoptosis were mediated by increasing Akt phosphorylation, whereas blocking Akt signaling effectively abolished the protective role of MT1/2 in ER stress and apoptosis (65). Furthermore, the phosphoinositide 3-kinase (PI3K)/protein kinase B (Akt) pathway is also essential for the tumor-suppressive role of MT1G in thyroid cancer. MT1G-suppressed Akt activation promotes Mdm2 upregulation and E-cadherin-mediated cell–cell adhesion suppression, both of which contribute to thyroid carcinogenesis (66). These results indicate that activation of the Akt signaling pathway is not only beneficial for MT1 to resist ER stress but also helpful for MT1 to restrain thyroid carcinogenesis (**Figure 2**).

A recent The Cancer Genome Atlas (TCGA) dataset and gene set enrichment analysis (GSEA) found that Wnt/β-catenin pathway-related genes were significantly enriched in hepatocellular cancer (HCC) patients with low MT1H expression, suggesting a potential association between the Wnt/β-catenin pathway and MT1H. Of note, ectopic overexpression of MT1H in HepG2 and Hep3B cells can suppress Wnt/β-catenin target genes *via* limiting nuclear translocation of β-catenin. The inhibition of the Wnt/β-catenin singling pathway by MT1H plays a critical antiproliferative and anti-invasive role in HCC, which eventually attenuates tumorigenicity (67). Collectively, these data indicate that Wnt/β-catenin signaling is a potent target downstream of MT1H in HCC (**Figure 2**).

## MT1 in Inflammatory Diseases

### Rheumatoid Arthritis

**Figure 3:**
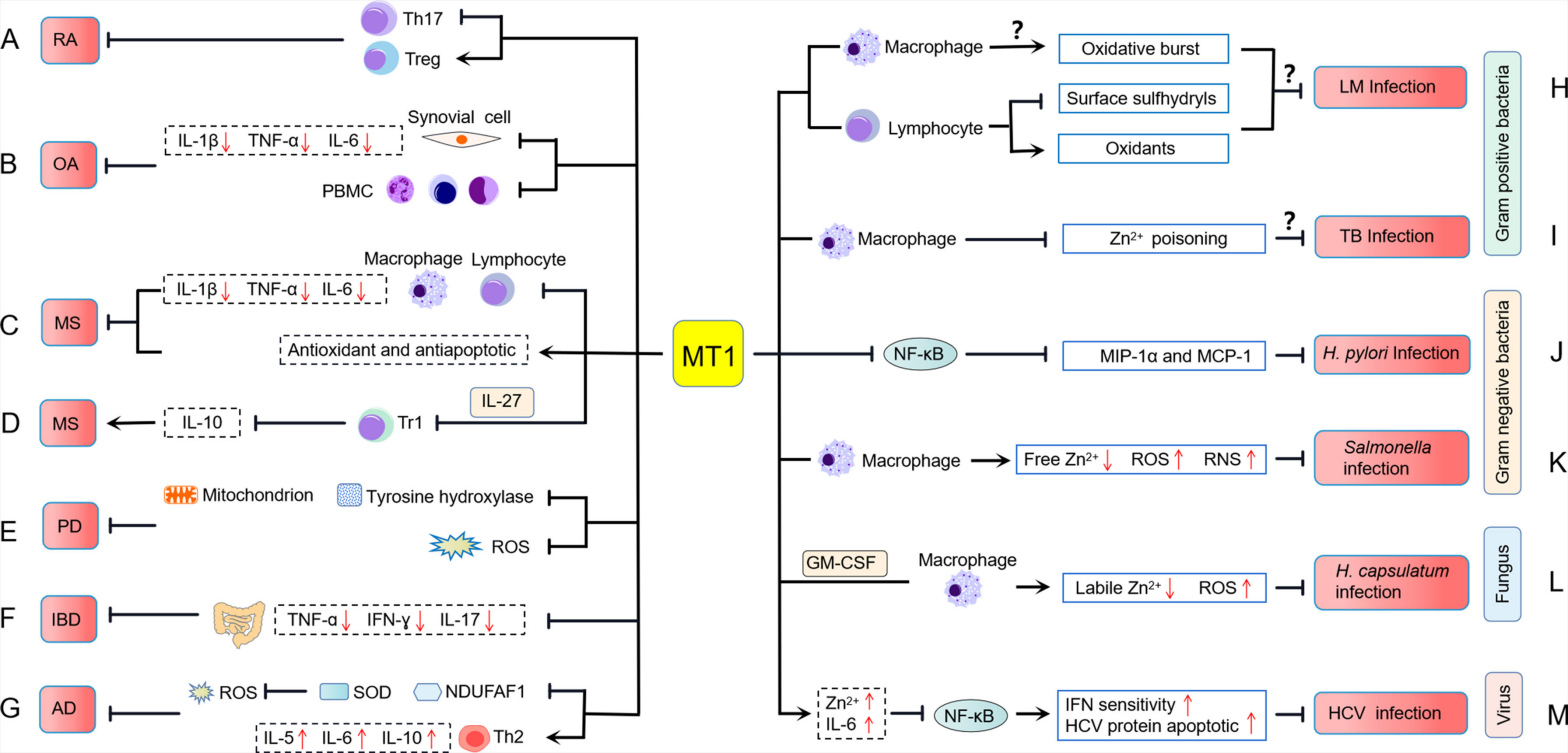
MT1 is involved in the pathology of inflammatory diseases. **(A)** MT1 inhibits rheumatoid arthritis (RA) pathogenesis by altering the balance of Th17/Treg cell immune homeostasis. **(B)** MT1 plays an anti-inflammatory role by inhibiting the expression of IL-1β, TNF-α, and IL-6 in PBMCs and synovial cells of erosive inflammatory osteoarthritis (EIOA) patients with an unclear molecular mechanism. **(C)** In multiple sclerosis (MS), MT1 suppresses the invasion of macrophages and lymphocytes into the CNS, downregulates the expression of proinflammatory cytokines, and increases the antioxidant and antiapoptotic capabilities of the host. **(D)** MT1 can promote the occurrence of MS by inhibiting the expression of IL-10 in IL-27-induced Tr1 cells. **(E)** MT1 plays a neuroprotective role in Parkinson’s disease (PD) through upregulating tyrosine hydroxylase expression, mitochondrial activity, and reducing ROS production. **(F)** MT1 restrains inflammatory bowel diseases (IBD) by suppressing proinflammatory cytokines production. **(G)** MT1 plays a protective role against atopic dermatitis (AD) development, which may rely on a SOD and NDUFAF1 mediated antioxidant mechanism and is accompanied by the upregulation of Th2-related cytokines. **(H)** MT1 influences the oxidative burst in macrophages and the surface sulfhydryls and oxidants of lymphocytes upon *Listeria monocytogenes* (LM) infection, but the exact mechanism of MT1 modulating host defenses against LM infection is not clear. **(I)** MT1 may negatively regulate the occurrence of *Mycobacterium tuberculosis* (TB) infection by preventing zinc poisoning in macrophages. **(J)** MT1 can prevent *Helicobacter pylori* (*H. pylori*)-induced pathological lesions by inhibiting NF-κB mediated MIP-1α and MCP-1 expressions. **(K)** MT1 restrains *Salmonella* infections by suppressing free zinc levels and promoting the production of ROS and RNS. **(L)** MT1 controls *Histoplasma capsulatum* (*H. capsulatum*) infection in a manner that decreases labile Zn^2+^ and increases ROS in GM-CSF-induced macrophages. **(M)** MT1 is implicated in the pathogenesis of hepatitis C virus (HCV) infection, which may be related to MT increasing zinc and IL-6 levels, leading to increased HCV protein apoptosis and IFN sensitivity by blocking NF-κB activation.

Rheumatoid arthritis (RA), one of the most common autoimmune diseases, is characterized by chronic inflammation of joints and surrounding tissues (68). Although the cause of RA is still unclear, it is believed to involve a disruption of immune homeostasis. As a stress response protein to sequester toxicants, MT has been shown to have functional effects on immune cells, indicating that MT1 may play a role in regulating autoimmune diseases such as RA (**Figure 3A**). Our group and other studies found that MT1 expression is significantly upregulated in rheumatoid arthritis and is closely related to RA disease (69, 70). Furthermore, the synovial inflammation and pathological symptoms in rheumatic mice were dramatically suppressed when MT1 was locally administrated (50, 69, 70). Further investigation revealed that MT1 inhibits RA pathogenesis by shifting the differentiation of CD4^+^ T cells toward Treg cells and reducing the frequency of Th17 cells (50). MT1 modulated the balance of Th17/Treg cell immune homeostasis in RA pathogenesis, most likely dependent on the activation of the STAT3 signaling pathway (50). In addition, MT1 may also play a critical role in regulating Th1 immunity in autoimmune arthritis (70, 71). According to these findings, MT1 may prove to be a potential therapeutic target for autoimmune diseases.

### Osteoarthritis

Osteoarthritis (OA) is a predominant degenerative disease characterized by synovial inflammation and cartilage destruction (72–76). Based on the clinical symptoms and inflammatory response grade, OA was classified into two types: primary generalized osteoarthritis (PGOA) and erosive inflammatory osteoarthritis (EIOA) (77). MT1 has recently been confirmed to be closely related to the development and pathogenies of OA. Elevated levels of MT1 expression were observed in EIOA patients compared with those of PGOA patients and HCs and were positively correlated with visual analog scale (VAS) score, C-reactive protein (CRP), and erythrocyte sedimentation rate (ESR). Furthermore, positive correlations between MT1 and IL-1β, TNF-α, or IL-6 in synovial cells were found, indicating that MT1 may be involved in the inflammatory regulation of OA. *In vitro*, MT1 demonstrated a prominent anti-inflammatory role by inhibiting the expression of IL-1β, TNF-α, and IL-6 in peripheral blood mononuclear cells (PBMCs) and synovial cells from EIOA patients (78) (**Figure 3B**). Despite the molecular mechanism of MT1 as a suppressor of OA is not clear, these findings suggest that MT1 might potentially become a novel therapeutic target for OA treatment in the future.

### Multiple Sclerosis

Multiple sclerosis (MS) is an inflammatory demyelinating disease of the central nervous system (CNS) with multifactorial pathogenesis. MT is thought to play an important role in the pathogenesis and progression of MS. Clinical data revealed that MT1 and MT2 expression levels were remarkably increased in the brain lesions of MS patients, and these two MT isoforms appear to be primarily present in macrophages/microglia and reactive astrocytes (79). Interestingly, inactive MS lesions had slightly higher MT expression than active MS lesions, indicating that MT may be involved in MS disease remission (79). Inflammatory cytokines such as IL-6 and TFN-α and oxidative stress found in MS lesions were most likely responsible for MT induction (80–82). These results are consistent with previous research on MT1 and MT2 expression during experimental autoimmune encephalomyelitis (EAE), a relevant preclinical animal model of MS (21, 83–90). Therefore, the expression profile of MT1 and MT2 might be a useful predictor of clinical signs of MS.

Accumulating research has stressed the beneficial effects of MT1 and MT2 in EAE using genetic MT1/2 knockout mice, preventing demyelination, enhancing neuroprotective capacities, and restraining inflammatory responses (83, 88, 91–93) (**Figure 3C**). In EAE, mice with MT1/2 deficiency showed increased macrophage and T-cell infiltration in the central nervous system (CNS) and elevated expression of proinflammatory cytokines IL-1β, IL-6, and TNF-α (83, 91, 92). These proinflammatory cytokines can further activate macrophages and lymphocytes to increase inflammation in the CNS (94). The loss of MT1 and MT2 also increases oxidative stress, which is a vital mediator of apoptotic cell death and myelin damage in EAE (86, 93, 95). Confirming these results, treatment with MT protein can ameliorate pathological progression in EAE (86, 96, 97). Overexpression of MT1 significantly reduces tissue loss and vascular edema while improving focal cerebral ischemia and reperfusion (98). Mice treated with MT2 showed decreased clinical symptoms, mortality, and inflammation of the CNS during EAE (96). Exogenous MT1 and MT2 also show antioxidant and antiapoptotic effects, which are beneficial for preventing demyelination and reducing axonal damage in EAE/MS (86, 97). Although MT1 and MT2 are considered physiologically equivalent, a recent study reveals their different biological effects in the EAE model. In the mouse EAE model, MT2 mainly improved clinical symptomatology and promoted disease remission, whereas MT1 had little impact on these outcomes. Surprisingly, MT1 had a general inhibitory role in regulating serum cytokine profiles, whereas MT2 seems to favor inducing splenic Th2 responses (84). Given that the metal (such as Zn and Cu)-binding abilities of MT1 and MT2 were not completely equivalent (99), more research into the binding ability of Zn/Cu with MT1 and MT2, and the release degree of Zn/Cu, may be required to answer their different biological characteristics in EAE.

Type 1 regulatory T cell (Tr1 cell) has been identified as a novel T regulatory cell (Treg) that controls inflammation in EAE by producing IL-10 (100). IL-27 can enhance the immunosuppressive role of Tr1 cells in the development of EAE by activating the STAT1/3 signaling pathway. A recent study indicates that MT1/2 can negatively regulate IL-10 production in human and mouse Tr1 cells and dampen IL-27-induced Tr1 cells. Moreover, the induction of IL-10 in developing Tr1 cells coincided with the delayed expression of MT1/2 (48). Adoptively transferring Tr1 cells deficient in MT1/2 can suppress EAE development by inducing IL-10 production and T-cell proliferation (48), contradicting previous reports that MTs play an anti-inflammatory role in the EAE model (86, 96, 97) (**Figure 3D**). These results indicate that MTs may have distinct immunological functions when responding to different cells and tissues. It is also essential to assess the precise effects of the MT1 and MT2 individually in specific situations to identify their dual-functional features in MS. The creation of MT1 and MT2 single knockout mice may provide a practical approach to solve this issue.

### Parkinson’s Disease

Parkinson’s disease (PD) is one of the most common inflammatory neurodegenerative diseases. It results from the progressive degeneration of dopamine neurons that innervate the striatum (101). MTs are metal-binding proteins in the CNS that are released by astrocytes and associated with neuroprotection. There is a substantial increase in the expression of MT1 isoforms, MT1E, MT1F, MT1G, MT1H, MT1M, and MT1X in both PD nigra and frontal cortex (102). Astrocytes play a neuroprotective role by upregulating the expression of MT1 (102), which indicates the importance of MT1 in the development of PD. The protective effect of MT1 in PD was recently identified through an artificial transducing experiment, in which human MT1A was transduced into mitochondria by a cell-penetrating artificial mitochondria-targeting peptide (CAMP) (103). Treating a cell culture model of PD with CAMP-hMT1A restored tyrosine hydroxylase expression and mitochondrial activity and reduced ROS production. Furthermore, injecting CAMP-hMT1A into a PD mouse model brain rescued movement impairment and dopaminergic neuronal degeneration (103). Therefore, delivery of MT1 into mitochondria might be therapeutic against PD by alleviating mitochondrial damage (**Figure 3E**).

### Inflammatory Bowel Diseases

Inflammatory bowel diseases (IBDs) such as ulcerative colitis and Crohn’s disease are known as refractory and recurrent gastrointestinal tract diseases. Although the characteristics of IBD point to a variety of possible causes, including genetic, infectious, and immunological factors, the precise pathogenesis of IBD remains unknown (104–106). The expression of MT1/2 was significantly higher in the inflamed colitis tissue from DSS-induced colitis mice. In the DSS-induced colitis mice, MT1/2 deficiency leads to disease exacerbation. It promotes the development of excessive intestinal inflammation by upregulating inflammatory cytokines, including TNF-α, IFN-γ, and IL-17 (107). MT1 secretion is most likely derived from F4/80-positive macrophages in the intestinal mucosal, ensuring its anti-intestinal inflammation properties. Although MT1 signaling failed to influence the number of F4/80-positive cells, the proinflammatory function of these cells was effectively restrained following LPS stimulation (107). These findings indicate that MT1/2 plays a protective role against intestinal inflammation (**Figure 3F**). MT1-expressing macrophages might be a therapeutic candidate in IBD.

### Atopic Dermatitis

Atopic dermatitis (AD) is a chronic recurrent inflammatory skin disease characterized by eczematous skin lesions that typically appear at the predilection sites (108). Previous studies demonstrated that the levels of MT1 expression were increased in AD-like lesional skin at a very early time point, suggesting that MT1 production was a rapid immune response to inflammation and may play an essential role in AD (109, 110). In response to topical contact of dinitrofluorobenzene (DNFB) stimulation, increased expression of MT1 was identified in the nucleus where MT1 might protect DNFB-induced murine AD-like model (109). Because nuclear MT1 localization improves protection against oxidative stress and genomic damage (111), the function of MT1 in the nucleus may be required to protect AD development. Using MT1/2 knockout mice, researchers further evaluated the possible role of MT1 in the development of DNFB-induced AD. They found that MT1/2-deficient mice had more severe AD in comparison to wild-type mice (109). Moreover, MT1/2 deficiency had more CD4^+^ T cells and decreased typical Th2-dominated inflammation. MT1/2-deficient AD-like mice also showed increased expression of superoxide dismutase (SOD) and NADH dehydrogenase [ubiquinone] 1 alpha subcomplex assembly factor 1 (NDUFAF1) (109). Because both SOD and NDUFAF1 are required in cells to defend against reactive oxygen species (ROS) (112, 113), the protective role of MT1/2 in AD seems to rely on an antioxidant mechanism mediated by SOD and NDUFAF1 (109). Despite the potential mechanisms of MT1 in AD being not yet clear, these findings indicate that MT1/2 plays a protective role against AD development (**Figure 3G**).

### Infectious Diseases

Numerous studies have found that MTs, notably MT1 and MT2, are related to various infectious diseases. Here, we systematically reviewed the associations and biological functions of MT in bacterial, fungal, and viral infections.

*Listeria monocytogenes* (LM) is a Gram-positive bacterium pathogenic in all mammals, including mice and humans. Early bacterial killing requires the recruitment of neutrophils, macrophages, and NK cells to sites of infection and the production of cytokines and bactericidal oxidants (114). Coordination of both innate and adaptive immune responses is required for effective host clearance (84). MTs have been shown to influence immune activities (115). The MT1 doses dramatically influence host defenses against LM infection (**Figure 3H**). Compared to the wild-type C57BL/6J (B6-WT) strain, a congenic partner that carries a larger number of MT1 genes (B6-MTTGN) and a congenic strain in which both MT1 and MT2 are disrupted (B6-MTKO) both showed lower bacterial burdens 3 days post-inoculation. This difference was prominent in the first 48 h of infection, after which LM clearance occurred at comparable rates in all three strains (116). Lymphocytes from B6-MTKO mice exhibited increased cell death and increased levels of surface sulfhydryls compared to B6-WT and B6-MTTGN mice. Lymphocytes from B6-MTTGN mice had increased levels of intracellular oxidants compared to B6-WT and B6-MTKO mice. The oxidative burst by macrophages from infected B6-MTTGN and B6-MTKO mice was increased (116), suggesting one mechanism by which these strains might reduce the LM burden.

Other Gram-positive bacterial infections, such as *Mycobacterium tuberculosis*, have also been linked to MTs induction. Transcriptional profile analysis of *M. tuberculosis*-infected macrophages revealed that MT1H, MT1M, MT1X, and MT2A genes, metal-regulatory transcription factor (MTF1), and plasma membrane zinc exporter ZnT1/SLC30A1, were remarkably upregulated (117). Furthermore, MTF1 nuclear translocation and intracellular zinc release were rapidly increased in macrophages following *M. tuberculosis* infection (117), implying that the establishment of MTF1-induced MT1 and MT2 responses upon *M. tuberculosis* infection resulted in the dissociation of zinc from the intracellular Zn–MT complex to prevent zinc poisoning in macrophages (**Figure 3I**).

*Helicobacter pylori* belongs to Gram-negative bacteria that cause gastric inflammation and cancer in humans. The results of these illnesses are highly connected to immune cell infiltration at inflammatory sites, which produces a variety of inflammatory factors, including macrophage inflammatory protein (MIP)-1α and monocyte chemoattractant protein (MCP)-1. *H. pylori* infection can induce MT expression in the gastric mucosa of mice with OLA129 and C57BL/6 genetic backgrounds but not in C57BL/6 mice. Mice lacking MT1/2 were more sensitive to *H. pylori* infection than wild-type mice, and MT1/2 deficiency accelerates the accumulation of inflammatory cells toward inflammatory sites in the stomach, and bacterial loads (118, 119). The anti-inflammatory role of MT1/2 in *H. pylori* infection is enormously dependent on inhibiting NF-κB-mediated MIP-1α and MCP-1 expressions (118) (**Figure 3J**). Thus, inducing MT1/2 may have a therapeutic effect in the prevention of *H. pylori*-induced pathological lesions.

*Salmonella* is a Gram-negative bacterium of the family Enterobacteriaceae, which usually causes gastrointestinal illness and salmonellosis. *Salmonella* infection can induce MT/2 expression and increase free zinc levels in macrophages (120). However, genetic deletion of MT1/2 in macrophages resulted in increased free zinc levels and decreased ROS and reactive nitrogen species (RNS) production and promoted the survival of *Salmonella* (120). Expansion of cellular zinc levels impairs the killing capability of *Salmonella* by macrophages by suppressing NF-κB activation and downstream ROS and RNS production and proinflammatory cytokines (120, 121) (**Figure 3K**). These results indicated that MT1/2 might play an important role in coordinating zinc-dependent mechanisms in response to intracellular *Salmonella* infections.

*Histoplasma capsulatum* is an ascomycetous fungus that can cause pulmonary and disseminated histoplasmosis. *H. capsulatum* survival in host phagocytic cells can be influenced by the dynamic variations of Zn-chelating MTs (122). MT1 and MT2 are the primary isoforms in macrophages that sequester zinc after *H. capsulatum* infection and activation by GM-CSF (37). Host MT1 and MT2 depletion reduced GM-CSF-induced ROS production, consistent with the labile zinc pool, resulting in more incredible oxidative burst and *H. capsulatum* clearance (37, 122) (**Figure 3L**). Therefore, MT1 and MT2 might play a vital role in the resistance to the pathogen *H. capsulatum* invasion.

In addition to participating in bacterial and fungal infections, MT1 is also associated with viral infections. Human coxsackievirus B type 3 (CB3) infection induces MT expression in several organs, including the liver, kidneys, and spleen (123). Upregulation of MT is associated with the redistribution of cadmium (Cd) and copper (Cu) elements (123). The influenza A virus (IAV) causes acute infection of the upper respiratory tract and lungs, resulting in increased MT1/2 expression in those tissues. Following IAV infection, IL-6 is needed exclusively in the liver for MT induction, whereas glucocorticoids are required for MT1 induction in both the liver and lungs (36). It was revealed that the MT1 upstream promoter contains nearly all metal response elements, MLTE/ARE and STAT3. The glucocorticoid responsive element (GRE1) was discovered to be situated upstream of the MT2 gene in the liver in response to IAV infection (36). These results demonstrate that MT induction by influenza virus infection might follow distinct signaling pathways.

In contrast to IAV infection, liver MT production in hepatitis C virus (HCV)-infected individuals was substantially lower than in control specimens (124). Liver MT expression was further negatively associated with disease activity and liver fibrosis in HCV-infected patients (124). The downregulated hepatic MT in HCV-infected patients is most likely associated with low IL-6 levels and zinc insufficiency (124). Thus, elevated liver MT levels appear to protect against HCV infection. Previous studies have reported that MT controls zinc homeostasis and NF-κB activity and that inhibiting NF-κB activation by MT may reverse the antiapoptotic effect of HCV protein and enhance cell sensitivity to IFN therapy (3, 61, 125, 126). Consistent with this observation, blocking NF-κB signaling sensitizes cells to the proapoptotic activity of IFNs, and supplementing zinc improves the response to IFN treatment in HCV patients (127–129). All these results suggest that MT seems to be involved in resistance to the pathogenesis of HCV infection and sustained beneficial response to IFN therapy in HCV infection (**Figure 3M**).

## Summary

MT1 has been thought to be a metalloprotein that primarily balances metals to relieve heavy metal poisoning and reduce stress responses over the last few decades. However, increasing data suggest that MT1 has various immunomodulatory effects by regulating several signal transduction pathways (**Figures 1**, **2**). Work in inflammatory diseases has further revealed the potent anti-inflammatory properties of MT1 (**Figure 3**). Nevertheless, there are still many scientific questions to be further answered. First, whether MT1 is found extracellular or intracellular complicates determining its precise immunoregulatory function. Expanding research to identify the receptor and intracellular signaling of MT1 will aid in our understanding of how MT1 modulates immune responses to control inflammatory diseases. Second, it is still unclear how the different isoforms of MT1 are produced in each inflammatory disease condition and how different isoforms contribute to their biological function. Third, most studies are based on using MT-knockout mice to investigate the effects of MT, which cannot completely reflect the independent regulatory characteristics of MT1. Constructing MT1-deficient mice may provide a new way to precisely measure MT1 function *in vivo*. Finally, further investigations are needed to elucidate the molecular mechanisms of MT1 in inflammatory diseases to facilitate the development of therapeutics.

## Author Contributions

HD reviewed the literature, generated figures, and wrote the paper. LW reviewed the literature, revised the paper, and offered feedback on the draft manuscript. LL, and ZH offered feedback on the draft manuscript. LY designed the concept of the work, reviewed the literature, and wrote and edited the paper. All authors contributed to the article and approved the submitted version.

## Funding

This work was supported by grants from the National Nature Science Foundation of China (32170937), Shenzhen Science and Technology Program (RCBS20200714114958310 and 20200803131335002), Guangdong Medical Science and Technology Research Foundation (A2021336), Guangdong Basic and Applied Basic Research Foundation (2020A1515110410 and 2020A1515010917), and SZU Top Ranking Project (86000000210) to LY, and the Shenzhen Science and Technology Program (JCYJ20170818093720089) and Guangdong Medical Science and Technology Research Foundation (A2019415) to LL.

## Conflict of Interest

The authors declare that the research was conducted in the absence of any commercial or financial relationships that could be construed as a potential conflict of interest.

## Publisher’s Note

All claims expressed in this article are solely those of the authors and do not necessarily represent those of their affiliated organizations, or those of the publisher, the editors and the reviewers. Any product that may be evaluated in this article, or claim that may be made by its manufacturer, is not guaranteed or endorsed by the publisher.

## Glossary

**Table:**

APC	antigen-presenting cell
BMDCs	bone marrow-derived dendritic cells
moDCs	human monocyte-derived DCs
FOXP3	forkhead box p3
CD86	cluster of differentiation 86
MHC-II)	major histocompatibility class II
ILT3	immunoglobulin-like transcript 3
ZnCl_2_	zinc chloride
ZnSO_4_	zinc sulfate
PDL2	programmed death-ligand 2
CD103	cluster of differentiation 103
Treg cell	regulatory T cell
HepG2	human hepatocellular carcinoma
MTF1	metal regulatory transcription factor 1
Cu	copper
As	arsenic
Cr	chromium
Cd	cadmium
tBHQ	tert-butylhydroquinone
Hepa1c1c7	mouse hepatoma cell line
LPS	lipopolysaccharides
IL-1β	interleukin-1β
IL-6	interleukin-6
IL-12	interleukin-12
IL-17	interleukin-17
IL-10	interleukin-10
TNF-α	tumor necrosis factor alpha
IL-4	interleukin-4
TSLP	thymic stromal lymphopoietin
Zip6	zinc transporter 6
STAT	signal transducer and activator of transcription
MLTF	major late transcription factor
ARE	antioxidant response element
GRE	glucocorticoid responsive element
SP1	specificity protein 1
*H. capsulatum*	*Histoplasma capsulatum*
BHK cells	baby hamster kidney fibroblasts
GILZ	glucocorticoid-induced leucine zipper
DCs	dendritic cells
GM-CSF	granulocyte-macrophage colony-stimulating factor
Tr1 cell	type I regulatory T cell
nTreg	natural regulatory T cell
Th17	T helper 17 cells
FcεRI	high-affinity IgE receptor
BMBAs	bone marrow-derived basophils
NFAT	nuclear factor of activated T cell
RORγt	retinoic acid related orphan receptor-γt
NF-κB	nuclear factor-kappa B
MMP-9	matrix metallopeptidase 9
Zip8	zinc importer Slc39A8
Znt5	zinc exporter Slc30a5
ER	endoplasmic reticulum
IH	intermittent hypoxia
PI3K	phosphoinositide 3-kinase
Akt	protein kinase B
TCGA	The Cancer Genome Atlas
GSEA	gene set enrichment analysis
HCC	hepatocellular cancer
RA	rheumatoid arthritis
OA	osteoarthritis
PGOA	primary generalized osteoarthritis
EIOA	erosive inflammatory osteoarthritis
MS	multiple sclerosis
EAE	experimental autoimmune encephalomyelitis
CNS	central nervous system
PD	Parkinson’s disease
CAMP	cell-penetrating artificial mitochondria-targeting peptide
IBD	inflammatory bowel diseases
AD	atopic dermatitis
DNFB	dinitrofluorobenzene
SOD	superoxide dismutase
NDUFAF1	NADH dehydrogenase s[ubiquinone] 1 alpha subcomplex assembly factor 1
ROS	reactive oxygen species
LM	*Listeria monocytogenes*
*H. pylori*	*Helicobacter pylori*
MIP-1α	macrophage inflammatory protein-1α
MCP-1	monocyte chemoattractant protein-1
RNS	reactive nitrogen species
*H. capsulatum*	*Histoplasma capsulatum*
CB3	human coxsackievirus B type 3
IAV	influenza A virus
HCV	hepatitis C virus
